# 2-Ethoxystypandrone, a novel small-molecule STAT3 signaling inhibitor from *Polygonum cuspidatum,* inhibits cell growth and induces apoptosis of HCC cells and HCC Cancer stem cells

**DOI:** 10.1186/s12906-019-2440-9

**Published:** 2019-02-01

**Authors:** Wuguo Li, Qing Zhang, Kaotan Chen, Zhenhua Sima, Jingli Liu, Qiang Yu, Jiawei Liu

**Affiliations:** 1 Ministry of Education Key Laboratory of Chinese Medicinal Resource from Lingnan, Research Center of Medicinal Plant Resource Science and Engineering, Guangzhou University of Chinese Medicine, 232 Waihuandong Road, Higher Education Mega Center, Guangzhou, 510006 China; 2 Departments of Pharmacology, , Shanghai Institute of Materia Medica, Chinese Academy of Sciences, 555 Zu Chong Zhi Rd., Zhangjiang Hi-Tech Park, Shanghai, 201203 People’s Republic of China; 3 Animal Experiment Center, The First Affiliated Hospital of Sun Yat-Sen University, Guangzhou, People’s Republic of China

**Keywords:** *Polygonum cuspidatum*, 2-Ethoxystypandrone, Hepatocellular carcinoma, Cancer stem cells, STAT3 signaling pathway, Cancer stemness inhibitor

## Abstract

**Background:**

Signal transducer and activator of transcription 3 (STAT3) is an oncogene constitutively activated in hepatocellular carcinoma (HCC) cells and HCC cancer stem cells (CSCs). Constitutively activated STAT3 plays a pivotal role in holding cancer stemness of HCC CSCs, which are essential for hepatoma initiation, relapse, metastasis and drug resistance. Therefore, STAT3 has been validated as a novel anti-cancer drug target and the strategies targeting HCC CSCs may bring new hopes to HCC therapy. This study aimed to isolate and identify small-molecule STAT3 signaling inhibitors targeting CSCs from the ethyl acetate (EtOAc) extract of the roots of *Polygonum cuspidatum* and to evaluate their in vitro anti-cancer activities.

**Methods:**

The chemical components of the EtOAc extract and the subfractions of *P. cuspidatum* were isolated by using various column chromatographies on silical gel, Sephadex LH-20, and preparative HPLC. Their chemical structures were then determined on the basis of spectroscopic data including NMR, MS and IR analysis and their physicochemical properties. The inhibitory effects of the isolated compounds against STAT3 signaling were screened by a STAT3-dependent luciferase reporter gene assay. The tyrosine phosphorylation of STAT3 was examined by Western Blot analysis. In vitro anti-cancer effects of the STAT3 pathway inhibitor were further evaluated on cell growth of human HCC cells by a MTT assay, on self-renewal capacity of HCC CSCs by the tumorsphere formation assay, and on cell cycle and apoptosis by flow cytometry analysis, respectively.

**Results:**

The EtOAc extract of the roots of *P. cuspidatum* was investigated and a novel juglone analogue 2-ethoxystypandrone (1) along with seven known compounds (2–8) was isolated. Among the eight isolated compounds 1–8, 2-ethoxystypandrone was a novel and potent STAT3 signaling inhibitor (IC_50_ = 7.75 ± 0.18 μM), and inhibited the IL-6-induced and constitutive activation of phosphorylation of STAT3 in HCC cells. Moreover, 2-ethoxystypandrone inhibited cell survival of HCC cells (IC_50_ = 3.69 ± 0.51 μM ~ 20.36 ± 2.90 μM), blocked the tumorspheres formation (IC_50_ = 2.70 ± 0.28 μM), and induced apoptosis of HCC CSCs in a dose-dependent manner.

**Conclusion:**

A novel juglone analogue 2-ethoxystypandrone was identified from the EtOAc extract of the roots of *P. cuspidatum* and was demonstrated to be a potent small-molecule STAT3 signaling inhibitor, which strongly blocked STAT3 activation, inhibited proliferation, and induced cell apoptosis of HCC cells and HCC CSCs. 2-Ethoxystypandrone as a STAT3 signaling inhibitor might be a promising lead compound for further development into an anti-CSCs drug.

**Electronic supplementary material:**

The online version of this article (10.1186/s12906-019-2440-9) contains supplementary material, which is available to authorized users.

## Background

Hepatocellular carcinoma (HCC), also called hepatoma, is the sixth most common cancer and the second leading cause of cancer-related death worldwide [1]. Despite advances in its treatment, the existing clinic therapies for HCC remain limited and its prognosis is very poor due to the high recurrence rate and the resistance to chemotherapy. Over recent years, emerging studies have demonstrated that these pathological properties are driven by HCC cancer stem cells (CSCs) reside among other more differentiated hepatoma cells [2–4], and HCC CSCs are responsible for cancer initiation, relapse, metastasis and drug resistance [5]. Therefore, HCC CSCs have become an important target for development of novel anti-cancer drugs. Development of therapeutic strategies targeting CSCs is urgently needed and may bring new hopes to treat HCC [6].

STAT3 is a signal transducer and transcription factor that regulates important cellular processes such as proliferation, differentiation, and apoptosis [7]. STAT3 is transiently activated to maintain homeostasis in normal liver cells. However, STAT3 is an oncogene constitutively activated in human HCC cells [8, 9]. Constitutively activated STAT3 also plays a pivotal role in holding cancer stemness of HCC CSCs [10, 11]. Therefore, STAT3 has been validated as anti-cancer drug target and targeting STAT3 signaling is considered as a novel promising therapeutic strategy for eradication of HCC CSCs [12, 13]. Identification of novel small-molecule STAT3 inhibitors from natural products will be of great interests in the development of novel anti-cancer therapeutics targeting HCC CSCs.

In searching for novel small-molecule STAT3 signaling inhibitors from medicinal plants, we have previously isolated a juglone analogue 2-methoxystypandrone and two anthraquinones from *Polygonum. cuspidatum* Sieb. et Zucc. as STAT3 signaling inhibitors [14] and found that 2-methoxystypandrone inhibited both STAT3 and NF-κB pathways dramatically by inhibiting Janus kinase 2 (JAK2) and IκB kinase (IKK) [15]. Juglone analogues have been isolated from numerous medicinal plants as active constituents, which exhibited many biological activities such as anti-viral, anti-bacterial, anti-inflammatory, and anti-cancer activities [16, 17]. Because of an interest in juglone analogues with STAT3 pathway inhibitory activities, the EtOAc extract of the roots of *P. cuspidatum* was re-examined and a novel juglone analogue 2-ethoxystypandrone (1) along with seven known compounds (2–8) were isolated. These isolated compounds were screened for their inhibitory effects on a STAT3 luciferase reporter gene in HepG2 cells. 2-Ethoxystypandrone (1) strongly blocked STAT3 activation (IC_50_ = 7.75 ± 0.18 μM) and inhibited the IL-6-induced as well as constitutive activation/phosphorylation of STAT3 in HCC cells. Moreover, 2-ethoxystypandrone (1) inhibited cell growth of HCC cells (IC_50_ = 3.69 ± 0.51 μM ~ 20.36 ± 2.90 μM), blocked the tumorspheres formation (IC_50_ = 2.70 ± 0.28 μM), and induced apoptosis of HCC CSCs in a dose-dependent manner.

## Methods

### General details

The ^1^H (400 and 500 MHz) and ^13^C NMR (100 and 125 MHz) spectra were determined on Avance 400 and Avance 500 Bruker spectrometers (Brucker, Germany). The chemical shifts were expressed in ppm as δ values relative to tetramethylsilane (TMS) as an internal standard. Mass spectra were recorded on DSQ ESI-mass spectrometer (Thermo, USA) and LC-MS-IT-TOF-mass spectrometer (Shimadzu, Japan). Analytical thin layer chromatography (TLC) was performed on silica gel 60 and visualized using Camag TLC visualizer by UV at 254 and 366 nm. Column chromatography was carried out on silica gel (Qindao Marine Chemical, China). Analytical HPLC was performed on a Agilent 1200 HPLC system (Agilent, USA) equipped with C_18_ column (250 × 4.5 mm i.d. stainless steel, 10 μm; Waters, USA); Preparative HPLC was performed on a Elite P270 HPLC system (Elite, China) equipped with C_18_ column (150 × 30 mm i.d. stainless steel, 10 μm; Waters). CombiFlash Rf200 flash chromatography performance (Teledyne ISCO, USA) was carried out on silica gel chromatography (40–60 μm, 4.1 × 23.5 cm, 120 g; Agela Technologies, China).

### Plant material

The roots of *Polygonum cuspidatum* (Polygonaceae) were purchased from Guangzhou Zhixing Pharmaceutical Co. Ltd. in 2011. Identification of the plant samples was verified by Dr. Guangtian Peng (Pharmaceutical School, Guangzhou University of Chinese Medicine). A voucher specimen (PC091101) of these materials was deposited for reference in the Research Center of Medicinal Plants Resource Science and Engineering, Guangzhou University of Chinese Medicine. The samples were stored in the shade at room temperature and pulverized before use.

### Extraction and isolation

The powdered roots of *P. cuspidatum* (1000 g) were extracted with EtOAc and MeOH using an E-914 speed extractor (Buchi, Swiss). The extracts were evaporated under vacuum to give the EtOAc (31.6 g) and MeOH (86.4 g) extracts, respectively. The EtOAc extract (11 g) was submitted to CombiFlash Rf-200 flash chromatography performance (Teledyne ISCO, USA) on silica gel column (40–60 μm, 3.7 × 23.5 cm, 120 g; Agela Technologies) and eluted with a step gradient of petroleum ether-ethyl acetate (PE-EtOAc) [100:0 (500 mL) → 0:100 (500 mL)] and EtOAc-MeOH [100:0 (500 mL) → 0:100 (500 mL)] to obtain 27 subfractions based on the TLC profile. Fractions Fr.5-Fr.9, which may contain 2-methoxystypandrone, emodin and physcion by monitoring with comparative TLC analysis, were not further investigated. The isolation methods of these active constituents in fractions were described in detail in previous studies [14]. Fractions Fr.4, Fr.10-Fr.11, Fr.14 and Fr.16-Fr.18 were subjected to additional chromatography. Fr.4 (1800 mL, 311 mg) was then applied to a silica gel column (25–40 μm, 4.0 × 30 cm) chromatography, eluting with a gradient of PE-EtOAc [98:2 (500 mL) → 60:40 (500 mL)] as solvent to give four subfractions. Sephadex LH-20 column was carried out to isolate Fr.4.3 (500 mL, 203 mg) with EtOH as eluting solvent to give two subfractions. Fr.4.3.2 (300 mL, 58 mg) was performed on a preparative C_18_ column (150 × 30 mm i.d. stainless steel, 10 μm; Waters) eluting with a stepwise gradient of MeOH-H_2_O (65:35 → 100:0) at 16 mL/min to yield 2-ethoxystypandrone (1, 5 mg, t_R_ = 11.0 min, 0.0014%). Fr.10 (600 mL, 106 mg) was separated by a preparative C_18_ column (150 × 30 mm i.d. stainless steel, 10 μm; Waters) eluting with a stepwise gradient of MeOH-H_2_O (34:66 → 100:0) at 18 mL/min to give Fr.10.3 (7.2 mg, t_R_ = 6.9 min) and citreorosein (2, 4 mg, t_R_ = 9.0 min, 0.0011%). Further separation of Fr.10.3 was chromatographed over a Sephadex LH-20 column using MeOH as eluting solvent to afford 4,6-dihydroxybenzofuran-3-one (3, 7 mg, 0.0020%). Fr.11 (500 mL, 294 mg), Fr.16 (660 mL, 237 mg) and Fr.18 (200 mL, 445 mg) were applied respectively to a preparative C_18_ column (150 × 30 mm i.d. stainless steel, 10 μm; Waters) eluting with a stepwise gradient of MeOH-H_2_O (30:70 → 100:0) at 16 mL/min to obtain resveratrol (4, 67 mg, t_R_ = 16.0 min, 0.0192%), polydatin-2′-O-gallate (5, 30 mg, t_R_ = 13.5 min, 0.0086%) and polydatin (6, 48 mg, t_R_ = 19.0 min, 0.0138%). Fr.14 (300 mL, 358 mg) was separated by a Sephadex LH-20 column using MeOH (1500 mL) as eluting solvent resulting in the isolation of catechin-3-O-gallate (7, 15 mg, 0.0043%). Fr.17 (360 mL, 427 mg) was chromatographed over a Sephadex LH-20 column using MeOH (1000 mL) as eluting solvent to result in forty sub-fractions and further separation of the sub-fraction Fr.17.6 (150 mL, 44 mg) was finished by the same chromatographic column to give 48 sub-fractions. Then sub-fraction Fr.17.6.3 (100 mL, 20 mg) was separated by a silica gel column (25–40 μm, 3.2 × 7 cm) chromatography eluting with a gradient of EtOAc-MeOH as solvent [100:0 (500 mL) → 50:50 (500 mL)] producing torachrysone-8-O-*β*-D-glucopyranoside (8, 8 mg, 0.0023%).

### Identification of 2-ethoxystypandrone (1)

2-Ethoxystypandrone (1): yellow acicular crystal (acetone); mp:153-154°C; UV (CH_2_Cl_2_): *λ*_max_: 227, 290, 408 nm; IR (KBr) *ν*_max_: 3064, 1686, 1632, 1592 cm^− 1^; ESI-MS: 275 [M + H]^+^; HR-ESI-MS: 273.0774 [M-H]^−^ (Calcd for C_15_H_14_O_5_: 273.0768); ^1^H NMR (400 MHz, acetone-*d*_6_): 1.48 (3H, t, *J* = 7.0 Hz, H-15), 2.35 (3H, s, H-13), 2.55 (3H, s, H-12), 4.22 (2H, q, *J* = 7.0 Hz, H-14), 6.25 (1H, s, H-3), 7.44 (1H, s, H-8); ^13^C NMR (100MHz, acetone-*d*_6_): 12.9 (C-15), 18.5 (C-13), 30.5 (C-12), 65.3 (C-14), 109.2 (C-3), 112.0 (C-10), 120.3 (C-8), 130.5 (C-6), 136.0 (C-9), 142.6 (C-7), 157.2 (C-5), 160.4 (C-2), 178.2 (C-1), 190.6 (C^− 4^), 201.6 (C-11).

### Cell lines and cell culture

Human hepatocellular carcinoma Hep3B and HepG2 were obtained from the American Type Culture Collection. Human hepatocellular carcinoma Huh-7, Li-7 and SK-HEP-1 cells were kindly provided by Stem Cell Bank, Chinese Academy of Sciences. HepG2/STAT3 cells were cultured in α-MEM (Life Technologies, USA) with 10% FCS (Life Technologies, USA) and the rest cell lines were cultured in DMEM (Life Technologies) with 10% FCS (Life Technologies, USA).

### Reporter plasmid construction and cell transfection

HepG2/STAT3 cells, a gift of Prof. Xinyuan Fu (National University of Singapore, Singapore), were the human hepatocellular carcinoma HepG2 cells stably transfected with a STAT3-responsive firefly luciferase reporter plasmid. Briefly, three tandem repeats of the oligonucleotide GATCGTCGACATTTCCCGTAAATC, which contains the DNA binding sequence of STAT3, was artificially synthesized and introduced into the luciferase reporter vector pGL2-P plasmid (Promega). The pGL2-P/STAT3 construct was confirmed by sequencing. The luciferase reporter plasmid pGL2-P/STAT3 was used to transfect HepG2 cells using Lipofectamine 2000 (Invitrogen). The stably transfected HepG2 cell line with the pGL2-P/STAT3 plasmid was obtained by screening for G418-resistant cell clones.

### STAT3-dependent luciferase reporter assay

The inhibitory activities on IL-6/STAT3 signaling were determined by STAT3-dependent luciferase reporter assay described in our previously report [14, 18]. HepG2/STAT3 cells were HepG2 cells stably transfected with a STAT3-responsive firefly luciferase reporter plasmid. The expression level of luciferase reached its peak value after 5–6 h stimulation by interleukin (IL)-6. HepG2/STAT3 cells (2 × 10^4^ per well) were seeded into 96-well cell culture microplates (Corning) and allowed to grow for 48 h and then treated with compounds 1–8 (0, 0.00001, 0.0001, 0.001, 0.01, 0.1, 1, 4, 20 μM) for 1 h followed by stimulation with 10 ng/mL interleukin (IL)-6 (BD Biosciences) for 5.5 h (cells were treated with compounds 1–8 for 6.5h). Luciferase activities were measured with the Promega luciferase kit (# E4550) according to the manufacturer’s instruction. All luciferase assay experiments were carried out at least thrice in triplicate to minimize the difference caused by cell number. The JAK2/STAT3 inhibitor pyridone 6 (Merck Chemical) was used as a positive control.

### Cell viability assay

The cell growth inhibitory activities on human hepatocellular carcinoma cells were examined by the MTT (3-(4,5-dimethylthiazol–2-yl)-2,5-diphenyl tetrazolium bromide) [18]. About 3.5 × 10^4^ cells per well were seeded into 96-well plates for 24 h. HepG2/STAT3 cells were then treated with vehicle control (DMSO) or 2-ethoxystypandrone (1) at indicated concentrations (0.016, 0.08, 0.4, 2, 10, 50, 100 μM) for 6.5 h. HCC Huh-7, Li-7, SK-HEP-1, HepG3B, and HepG2 cells were treated with 2-ethoxystypandrone (1) at indicated concentrations for 72 h. The assay was carried out by adding a MTT solution (20 μL, 5 mg/mL; Sigma Chemical Co.) to each well and incubating for 3 h at 37 °C to form MTT-formazan crystals by metabolically viable cells. The supernatant was aspirated, and the MTT-formazan crystals were dissolved in DMSO (150 μL). The absorbance was measured by a microplate reader (BioTek Instruments, USA) at a wavelength of 492/620 nm.

### HCC Cancer stem cells culture

HCC cancer stem cells were isolated from HCC Huh-7 cell line by serum-free cancer stem cell culture selection [19]. The Huh-7 cells were collected and washed to remove serum, then suspended and plated in cancer stem cell conditioned culture medium (CSC medium) supplemented with 100 IU/ML pennicillin and 100 μg/ml streptomycin. CSC medium used for sustaining cancer stemness was serum-free DMEM/F12 containing 10 ng/ml basic fibroblast growth factor (bFGF), 20 ng/ml epidermal growth factor (EGF), 4 μg/ml heparin, 100 μg/ml apotransferrin, 25 μg/ml insulin, 9.6 μg/ml putrescin, 20 nM progesterone, 30 nM sodium selenite anhydrous, and 4 mg/ml bovine serum albumin. The Huh-7 cells were subsequently cultured in ultra-low attachment 6-well plates at a density of 5000 cells/well, half volume of the flesh medium was exchanged every second day. After 10–14 days of culture, the most Huh-7 cells died and the cells grew up as nonadherent, three-dimensional sphere clusters.

### Tumorsphere passage and tumorsphere formation assay

To propagate the tumorspheres in vitro, spheres were collected by gentle centrifugation, dissociated with 0.005% trypsin-EDTA for 2 min, then added CSC medium and mechanically dissociated with a pipette to single cells. The resulting single cells were then centrifuged to remove the enzyme and re-suspended in CSC medium allowed to reform the tumorspheres. The spheres could be passaged every 7 days, and no more than 20 passages can be used for the tumorsphere formation assay. The dissociated single sphere-forming cells were diluted to a density of 1000 cells/ml, Then, the 1ml/well diluted cell suspension was plated to ultra-low attachment 12 wells plate and treated with indicated concentrations (0, 0.5, 1, 2, 3, 5, 8 and 10 μM) of compound 2-ethoxystypandrone for 72 h, the tumorspheres were photographed under an inverted microscope (Leica DMi8, Germany) and the tumorspheres larger than 50 μm in diameter were counted using ImageJ 1.45.

### Western blot analysis

Expression of STAT3 and p-STAT3 proteins was analyzed by Western Blot analysis described in previous report [18]. HepG2/STAT3 cells or HepG2 cells were grown to 70–80% confluency and then pre-treated with 2-ethoxystypandrone (1) at different concentrations (0, 2, 10, 50,100 μM) for 2 h. The cells were stimulated with IL-6 for 15 min (the phosphorylation of STAT3 appeared in the 15 min) and then lysed in Laemmli sample buffer and boiled for 5 min. Proteins were then separated by 8% SDS-PAGE and transferred to nitrocellulose membranes. Membranes were blocked in TBS containing 5% nonfat milk 0.1% and Tween 20 (TBST) for 1 h at room temperature and then incubated for 2 h in TBST containing primary antibodies and 5% bovine serum albumin. After being washed with TBST, membranes were then incubated with horseradish peroxidase-conjugated goat anti-mouse IgG (H + L) secondary antibody (#GAM007, MultiSciences) for 1 h, and finally immune complexes were detected by enhanced chemiluminescence. Primary antibodies used in Western blot were mouse anti-STAT3 (#9139,CST), mouse anti-pY-STAT3 (Tyrosine 705, #9138, CST), and mouse anti-α-tubulin (#sc5286, Santa Cruz).

### Flow cytometric analysis

Huh-7 cells were grown to 70–80% confluence and then treated with various concentrations (0, 2, 4, and 8 μM) of 2-ethoxystypandrone (1) for 24 h. The cells were harvested and fixed in 70% ethanol for 30 min at 4 °C. The cells were then treated with FITC Annexin V apoptosis detection kit I (BD Biosciences Pharmingen) according to the manufacturer’s protocol and the samples were immediately then analyzed by flow cytometry (BD Canto II, USA). The percentage of cells undergoing apoptosis was calculated with CELLQuest 3.0 software. The dots in the lower left quadrant, upper left quadrant, upper right quadrant, and lower right quadrant represent the viable cells, the damages cells, the cells in late apoptosis and the cells in early apoptosis, respectively.

For analysis the apoptosis of HCC CSCs, the suspended tumorspheres were harvested and dissociated mechanically into single cells with 0.005% trypsin-EDTA for 2 min and the dissociated cells were re-seeded with CSC medium and treated with indicated concentrations (0, 5, 8, and 10 μM) of 2-ethoxystypandrone (1) for 24 h. The cells were harvested and then treated with FITC Annexin V apoptosis detection kit I (BD Biosciences Pharmingen, USA) according to the manufacturer’s protocol respectively and the samples were immediately then analyzed by flow cytometry (BD Canto II, USA). In addition, for analysis the cell cycle of HCC CSCs, the suspended tumorspheres were harvested and dissociated mechanically into single cells with 0.005% trypsin-EDTA for 2 min and the dissociated cells were re-seeded with CSC medium and treated with indicated concentrations (0 and 10 μM) of 2-ethoxystypandrone (1) for 24 h. The cells were harvested and fixed in 70% ethanol for 2 h. After washing the cells with pre-cooled PBS twice, the cells were then treated with Propidium iodide (PI)/RNase Staining Buffer (BD Biosciences Pharmingen, USA) according to the manufacturer’s protocol respectively and the samples were immediately then analyzed by flow cytometry (BD Canto II,USA).

### Statistic analysis

All data were analyzed using GraphPad software (Graph-Pad Prism version 4.0 for windows) and presented as Mean ± standard error of the mean (SEM). IC_50_ values (50%concentration of inhibition) were determined through non-linear regression analysis. Three independent experiments were performed. SPSS 19.0 software was used for statistical analysis. *p < 0.05 and ***p < 0.001 indicate a statistically significant difference as compared to control group.

## Results

### Isolation and structure determination

**Fig. 1:**
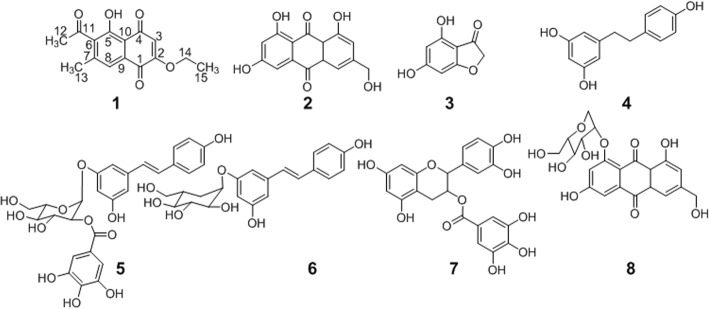
Chemical structures of compounds 1–8 isolated from *P.cuspidatum*

The EtOAc extract of roots of *P. cuspidatum* was subjected to repeated silica gel column chromatography followed by subsequent sephadex LH-20 column or/and preparative HPLC separation to give a novel compound (1) along with seven known compounds (2–8). Compound (1), a yellow acicular crystal in acetone, possessed a molecular formula of C_15_H_14_O_5_ as established by ESI-MS ([M + H]^+^ at *m/z* 274.98) (Additional file 1: Figure S2) and HR-ESI-MS ([M-H]^−^ at *m/z* 273.0774 (Calcd. 273.07678)) (Additional file 1: Figure S3). The UV spectrum showed absorption maxima characteristic of an α, β-unsaturated ketone chromophore at 408, 290 and 227 nm. The IR spectrum showed absorption bands due to C-H (3064 cm^− 1^) and carbonyls (1686, 1632, 1592 cm^− 1^)(Additional file 1: Figure S1). ^1^H NMR and ^1^H - ^1^H COSY NMR spectrum (Additional file 1: Figures S4 and S5) revealed signals of two aromatic protons at δ_H_ 7.44 (1H, s, H-8) and 6.25 (1H, s, H-3), two methyls at δ_H_ 2.55 (3H, s, H-12) and 2.35 (3H, s, H-13) and an oxethyl at δ_H_ 4.22 (2H, q, *J* = 7.0 Hz, H-14) and 1.48 (3H, t, *J* = 7.0 Hz, H-15). ^13^C NMR spectrum (Additional file 1: Figure S6) displayed the presence of 15 carbons, including three methyls (C-12, C-13 and C-15), one methylene (C-14), two methines (C-3 and C-8) and nine quaternary (C-1, C-2, C-4, C-5, C-6, C-7, C-9, C-10 and C-11). All protonated carbons and their bonded protons were determined by the HSQC (Additional file 1: Figure S8) and HMBC (Additional file 1: Figure S9). Aromatic protons at δ_H_ 6.25 and 7.44 were found to be correlated to C-3 at δ_C_ 109.2 and C-8 at δ_C_ 120.3 respectively in the HSQC spectrum. A series of HMBC correlations from H-8 to C-1, C-6, C-7, C-10 and C-13, from H-12 to C-6 and C-11, from H-13 to C-6, C-7 and C-8 and from H-14 to C-2 and C-15 were found (Table 1). The ^1^H NMR and ^13^C NMR signals of compound 1 were similar to those of 2-methoxystypandrone (2-methoxy-6-acetyl-7-methyl-juglone) except for an ethoxyl group substitution at C-2 [20]. Thus, the chemical structure of compound 1 was determined as 2-ethoxy-6-acetyl-7-methyl-juglone, named as 2-ethoxystypandrone. The known compounds (2–8) were respectively identified as: citreorosein (2) [21], 4,6-dihydroxybenzofuran-3-one (3) [22], resveratrol (4) [23], polydatin-2′-O-gallate (5), polydatin (6), catechin-3-O-gallate (7) [24] and torachrysone-8-O-β-D-glucopyranoside (8) [25] by comparing their spectroscopic data (^1^H, ^13^C NMR, and MS) with those reported in the literatures. The chemical structures of the isolated compounds 1–8 are shown in Fig. 1.

**Table 1:** ^1^H and ^13^C NMR data of 2-ethoxystypandrone (**1**)(^1^H 400MHz, ^13^C 100MHz in acetone-*d*_6_)

No	^13^C δ (ppm)	^1^H δ (ppm)	HMBC (^1^H → ^13^C)
1	178.2(C)		H-8; H-3
2	160.4(C)		H-3; H-14
3	109.2(CH)	6.25 (s)	
4	190.6(C)		H-3
5	157.2(C)		
6	130.5(C)		H-12; H-13; H-8
7	142.6(C)		H-8; H-13
8	120.3(CH)	7.44 (s)	H-13
9	136.0(C)		
10	112.0(C)		H-3; H-8
11	201.6(C)		H-12
12	30.5(CH_3_)	2.55 (s)	
13	18.5(CH_3_)	2.35 (s)	H-8
14	65.3(CH_2_)	4.22(q, *J* = 7.0 Hz)	H-15
15	12.9(CH_3_)	1.48(t, *J* = 7.0 Hz)	H-14

### Inhibitory activity of the isolated compounds against STAT3 signaling pathway

**Fig. 2:**
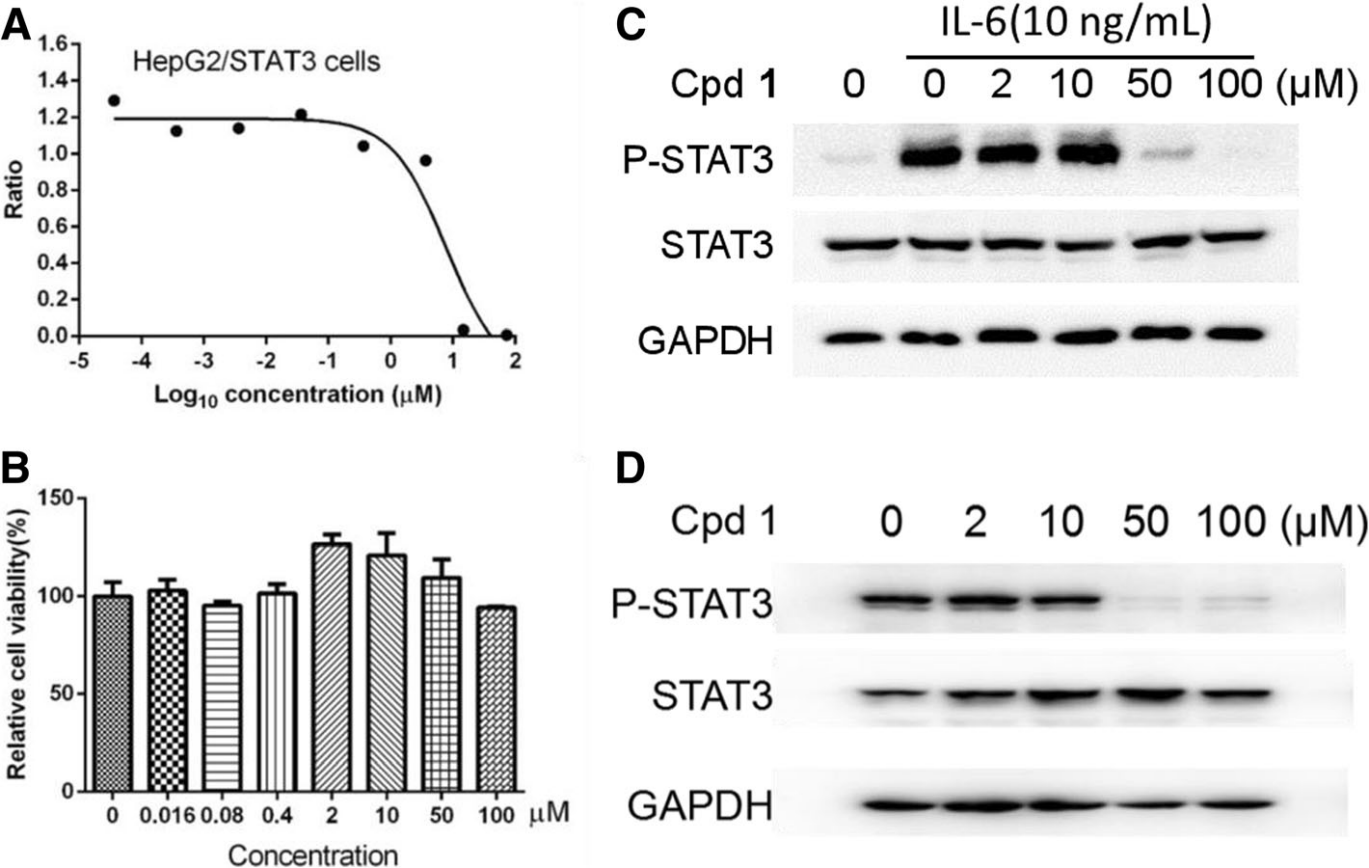
2-Ethoxystypandrone (1) inhibited the IL-6-induced and constitutive activation of STAT3. **a**. Dose-dependent inhibition of 2-ethoxystypandrone (1) on the IL-6-induced STAT3 activity. HepG2/STAT3-luciferase cells were pretreated with 2-ethoxystypandrone (1) at indicated concentrations for 1 h, and the luciferase activity was measured following stimulation of IL-6 (10 ng/mL) for 5.5 h; **b**. HepG2/STAT3-luciferase cells were treated with indicated concentrations of 2-ethoxystypandrone (1) for 6.5 h. Cell viability was determined by MTT assay; **c**. Compound 2-Ethoxystypandrone (Cpd 1) inhibited the IL-6-induced phosphorylation of STAT3 in HepG2/STAT3-luciferase cells. HepG2/STAT3 luciferase cells were pre-treated with 2-ethoxystypandrone (1) at the indicated concentrations for 2 h and were then stimulated with 10 ng/mL interleukin-6 for 15 min and the cell lysates were prepared for Western Blot analysis using anti-phospho-STAT3 antibody; **d**. 2-Ethoxystypandrone (1) inhibited the basal constitutive activation of STAT3 in HepG2 cells. The HepG2 cell lysates were prepared for Westerm Blot after cells were pre-treated with 2-ethoxystypandrone (1) for 2 h and without IL-6 treatment

The inhibitory effects of all the isolated compounds 1–8 on the IL-6/STAT3 signaling pathway were re-examined by a cellular STAT3 luciferase gene reporter assay. Pyridone 6, a known JAK2/STAT3 signaling inhibitor [26], was used as a positive control (IC_50_ = 0.02 ± 0.001 μM). The IC_50_ values of compounds 1–8 are compared in Table 2. As shown in Fig 2a and Tables 2, 2-ethoxystypandrone (1) was the most potent inhibitor among the evaluated compounds 1–8, and demonstrated a significant inhibitory effect on the STAT3 transcription activity with an IC_50_ value of 7.75 ± 0.18μM in a dose-dependent manner (Fig. 2a). 2-Ethoxystypandrone (1), which has ethoxyl (2-OCH_2_CH_3_) moiety at C-2, showed 2-fold increased potency compared with our previously reported compound 2-methoxystypandrone containing methoxyl (2-OCH_3_) group against STAT3 signaling with an IC_50_ value of 17.25 ± 0.21 μM [14]. Citreorosein (2) also exhibited considerable inhibitory activities against the IL-6/STAT3 signaling pathway (IC_50_ = 40.20 ± 0.17 μM). However, compounds 3–8, at the concentration of 20 μg/mL, did not exhibit any activities on the IL-6/STAT3 signaling pathway (Table 2). The HepG2/STAT3-luciferase cells were treated with indicated concentrations of 2-ethoxystypandrone (1) for 6.5 h and the MTT assay showed that the cells were not completely dead after the treatment of 2-ethoxystypandrone (1) for 6.5 h (Fig. 2b), suggesting that the inhibition of STAT3 phosphorylation was not caused by the cytotoxic effects of 2-ethoxystypandrone (1). To study the mechanism of action of 2-ethoxystypandrone (1) on STAT3 signaling, the tyrosine phosphorylation of STAT3 was examined by Western blot analysis. 2-Ethoxystypandrone (1) was found to inhibit the IL-6-induced STAT3 tyrosine phosphorylation in a dose-dependent manner (Fig. 2c). 2-Ethoxystypandrone (1) showed similar inhibitory effects on the basal activation of STAT3 in the HepG2 cells (Fig. 2d). Therefore, 2-ethoxystypandrone (1) could inhibit both the IL-6-induced as well as the basal activation of STAT3.

**Table 2:** Effects of compounds **1–8** on IL-6/STAT3 activity in HepG2/STAT3 cells

compound	**1**	**2**	**3**	**4**	**5**	**6**	**7**	**8**	pyridone 6
IC_50_ (μM)	7.75 ± 0.18	40.20 ± 0.17	> 100	> 100	> 100	> 100	> 100	> 100	0.02 ± 0.001

### 2-Ethoxystypandrone (1) inhibited cell growth and induced apoptosis of HCC cells

**Fig. 3:**
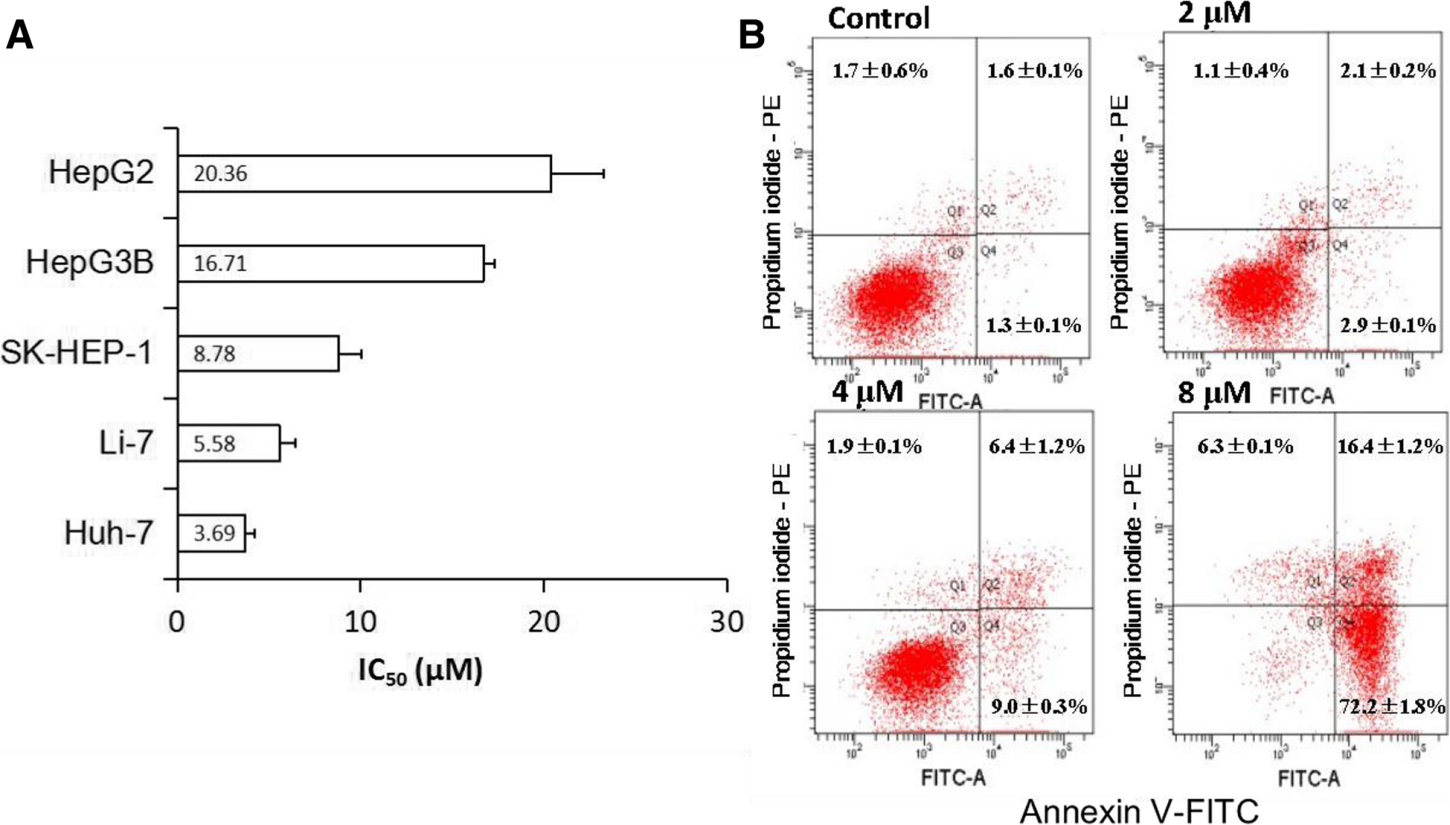
2-Ethoxystypandrone (1) inhibited cell proliferation and induced apoptosis of HCC cells. **a**. IC_50_ values of 2-ethoxystypandrone (1) on the viability of different human HCC cell lines. **b** HCC Huh-7 cells were treated with 2-ethoxystypandrone (1) at the indicated concentration for 24 h. After being stained with Annexin V-FITC and propidium iodide, cells were analyzed using flow cytometry. The percentage (%) of apoptotic cells was calculated with software

STAT3 is constitutively activated in human hepatocellular carcinoma (HCC) cells, and the inhibition of STAT3 signaling pathway affects cell growth and induces cell apoptosis of HCC cells [27, 28]. We examined whether 2-ethoxystypandrone (1) was effective in inhibiting cell growth and cell survival of human HCC cell lines (Huh-7, Li-7, SK-HEP-1, HepG3B, and HepG2) using the MTT assay. Different human HCC cells lines were treated with different doses of 2-ethoxystypandrone (1) for 72 h, and 2-ethoxystypandrone (1) inhibited the growth of all the tested HCC cells. The half maximal inhibitory concentration (IC_50_) values of 2-ethoxystypandrone (1) ranged from 3.69 ± 0.51 μM to 20.36 ± 2.90 μM (Fig. 3a). It was noted that the sensitivity of different HCC cells to 2-ethoxystypandrone (1) treatment varied. HCC Huh-7 and Li-7 cells with constitutively activated STAT3 [27] were more sensitive to 2-ethoxystypandrone (1) (IC_50_ = 3.69 ± 0.51 μM and 5.58 ± 0.89 μM, respectively) than the HepG3B and HepG2 cells (IC_50_ = 16.71 ± 0.58 μM and 20.36 ± 2.90 μM, respectively). To further understand the nature of the 2-ethoxystypandrone (1)-induced inhibition of cell growth, Huh-7 cells were randomly selected to examine the effects of 2-ethoxystypandrone (1) on the induction of cell apoptosis. Cell apoptosis was observed at 24 h after the inhibition of STAT3 activity, as indicated by the cleavage of poly (ADP-ribose) polymerase (PARP), which has been shown to occur as early as at 4 h after STAT3 inhibition but became more significantly at 24 h [29, 30]. HCC Huh-7 cells were treated with different doses of 2-ethoxystypandrone (1) for 24 h and stained with Annexin V /Propidium Iodide (PI). Flow cytometry analyses showed that 2-ethoxystypandrone (1) induced Huh-7 cells apoptosis in a concentration-dependent manner (Fig. 3b). The total apoptosis of was 88.6 ± 1.4% after 24 h treatment of 2-ethoxystypandrone (1) at 8 μM, while control group was 2.9 ± 0.6%. Treatment of 2-ethoxystypandrone (1) at 8 μM for 24 h resulted in higher population of late apoptotic cells (77.2 ± 1.8%) compared to the control (1.3 ± 0.1%). The data showed that 2-ethoxystypandrone (1) induced a dose-dependent apoptosis in Huh-7 cells at the later stage. Taken together, our data showed that 2-ethoxystypandrone (1) was able to inhibit cell growth and induce apoptosis of HCC cells, especially those with constitutively activated STAT3.

### Ethoxystypandrone (1) blocked self-renewal and induced cell-cycle arrest and apoptosis in HCC CSCs

**Fig. 4:**
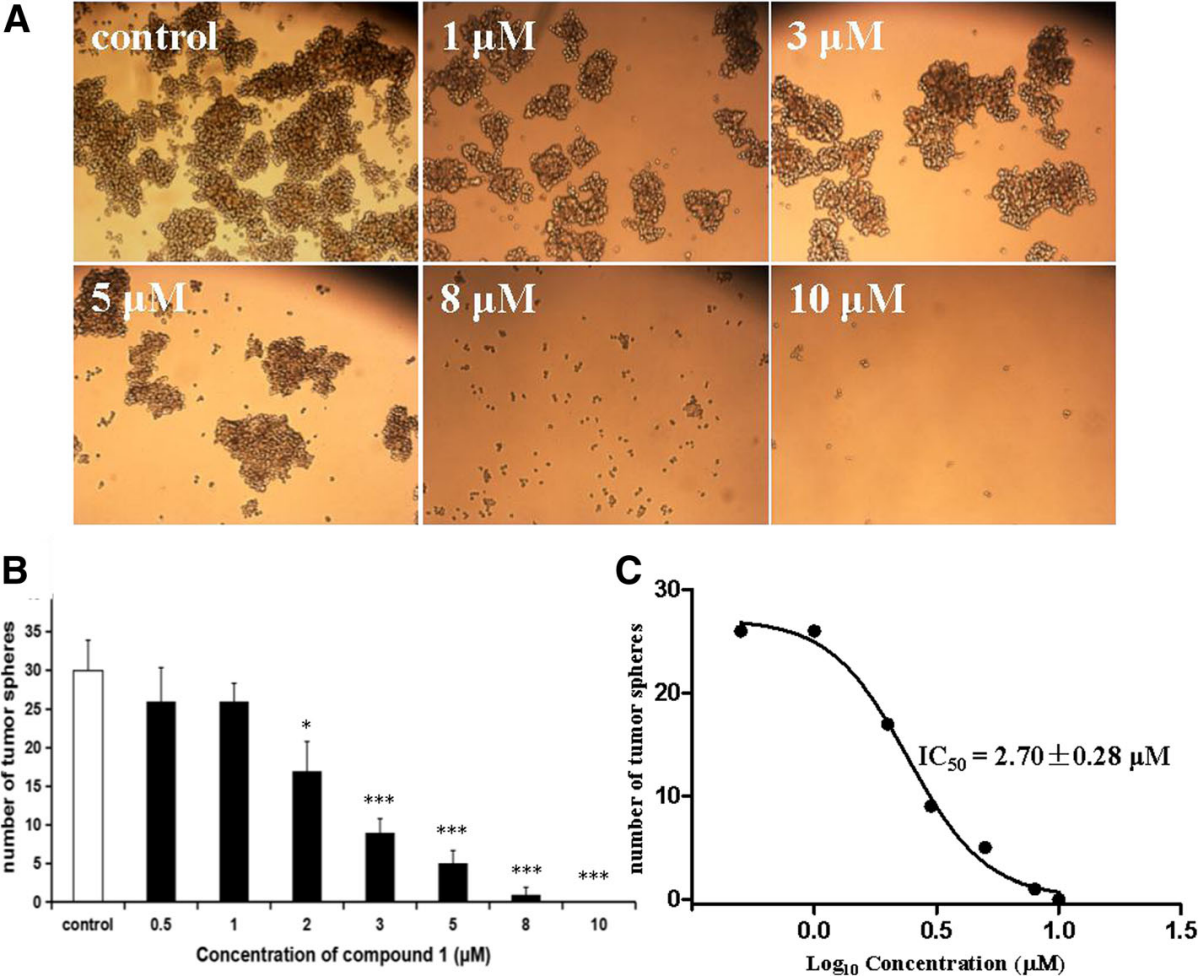
2-Ethoxystypandrone (1) inhibited self-renewal, cell growth and the tumorsphere formation of HCC CSCs in dose-dependent manner. **a**. HCC CSCs were treated with 2-ethoxystypandrone (1) at the indicated concentration for 72 h. **b**. Number of tumorspheres after being treated with 2-ethoxystypandrone (**1**) at the indicated concentration for 72 h. Values are means + SEM, *p < 0.05 and ***p < 0.001, compared to control group. One-way ANOVA was used for statistical analysis by SPSS 19.0 software. **c**. The growth curve of the tumorspheres after being treated 2-ethoxystypandrone (1) at the indicated concentration for 72 h

**Fig. 5:**
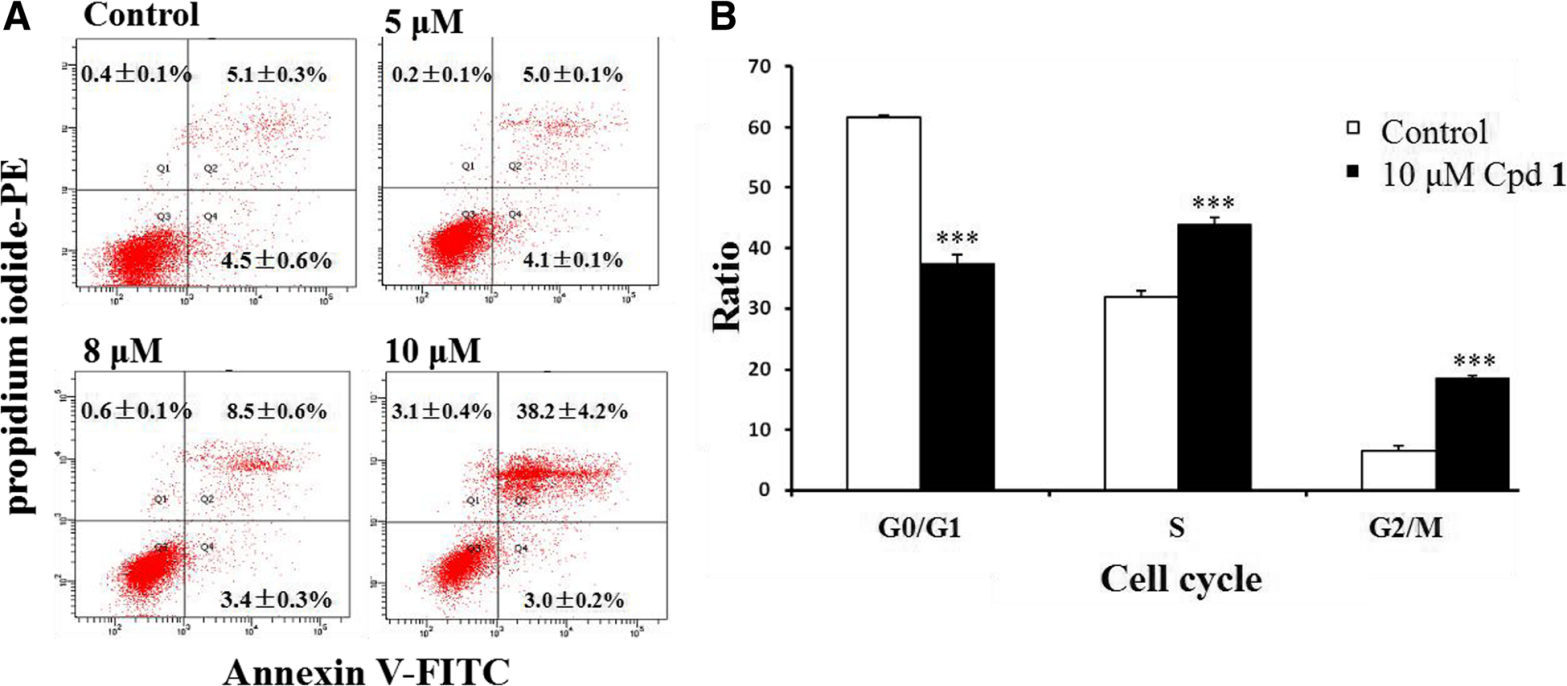
The analysis of cell apoptosis and cell cycle distribution of HCC CSCs induced by 2-ethoxystypandrone (1). **a**. 2-Ethoxystypandrone (1) induced cell apoptosis of HCC CSCs in dose-dependent manner. Apoptosis rates of HCC CSCs after being treated with 2-ethoxystypandrone (1) at the indicated concentration for 24 h. **b**. The cell cycle distribution of HCC CSCs after being treated with 2-ethoxystypandrone (1) at the concentration of 10 μM for 24 h. Values are means+SEM, *p < 0.05 and ***p < 0.001, compared to control group. Two-tailed unpaired T-Test was used for statistical analysis by SPSS 19.0 software

The HCC CSCs were isolated from HCC Huh-7 cells by stem cell culture selection and characterized by their in vitro CD133^+^ surface phenotype and greater ability to form hepatoma xenografts in vivo in our laboratory (unpublished data). HCC Huh-7 cells were cultured in a serum-free stem cell conditioned culture medium at in ultra-low attachment 6-well plates, which allowed for the formation of the non-adherent, three-dimensional sphere clusters (Fig. 4a). Tumorspheres were able to passage more than 20 generations and even single cell from spheres could be propagated to form new spheres again, indicating their in vitro self-renewal capability of HCC CSCs isolated from HCC Huh-7 cells in our laboratory. To further determine the effects of 2-ethoxystypandrone (1) on HCC CSCs, we assessed the self-renewal capability of HCC CSCs using the tumorsphere formation assay. After treatment with seven different concentrations (0.5, 1, 2, 3, 5, 8 and 10 μM) of 2-ethoxystypandrone (1) for 72 h, the tumorspheres were inhibited by 2-ethoxystypandrone (1) in a dose-dependent manner (Fig. 4b), 2-ethoxystypandrone (1) at the low concentration of 2 μM significantly blocked self-renewal and the tumorsphere formation of HCC CSCs compared with control group. The IC_50_ value of 2-ethoxystypandrone (1) was 2.70 ± 0.28 μM in the tumorsphere formation assay (Fig. 4c). In order to understand whether the inhibitory effect of 2-ethoxystypandrone (1) on the tumorsphere formation was due to cell-cycle arrest, we evaluated the effects of 2-ethoxystypandrone (1) treatment on cell-cycle distribution of HCC CSCs using PI staining and flow cytometry analysis. As shown in Fig. 5b, 61.65 ± 0.28% of cells in control group were in G0/G1, 31.86 ± 1.07% were in S phase, and 6.48 ± 0.86% were in G2/M at 24h, respectively. However, in the 2-ethoxystypandrone (1)-treated group, the relative number of cells in S phase was increased to 43.96 ± 1.07%, the number of cells in G2/M phase was also increased to 18.5 ± 0.36%, the number of cells in G0/G1 decreased to 37.53 ± 1.32%, respectively. 2-Ethoxystypandrone (1) significantly increased the S and G2/M phases cell population of HCC CSCs, accompanied with the decreased G0/G1 phase cell population compared to that of control group. These data suggested that 2-ethoxystypandrone (1) induced the cell-cycle arrest at the S and G2/M phases, then inhibited self-renewal and cell growth, and blocked the tumorspere formation of HCC CSCs. In addition, the cell growth inhibition could also be attributable to cell apoptosis of HCC CSCs. Hence, we further examined whether 2-ethoxystypandrone (1) could induce apoptosis of HCC CSCs using flow cytometric analysis. As shown in Fig. 5a, the percentage of apoptotic cells in control group was 9.6 ± 0.9%, and was significantly increased to 41.2 ± 4.3% in 2-ethoxystypandrone (1) at 10 μM for 24 h (Fig. 5a). 2-Ethoxystypandrone (1) exposure resulted in higher population (38.2 ± 4.2%) of early apoptotic cells in HCC CSCs (Fig. 5a). Collectively, these results indicated that 2-ethoxystypandrone (1) was able to inhibit self-renewal, block the tumorsphere formation and induce apoptosis of HCC CSCs by blocking cell-cycle progression at the S and G2/M phases.

## Discussion

Despite the progress in understanding carcinogenesis and therapeutic approaches, the prognosis of HCC patients remains poor, mainly due to its high recurrence rate and drug resistance. Emerging evidences have demonstrated that the highly resistant cancer stem cells (CSCs) possess stemness properties and play critical roles in cancer initiation, progression, relapse, metastasis, and chemoresistance [5, 31]. The cancer stemness properties are governed by many oncogenic pathways such as STAT3, NOTCH, WNT, and NANOG, which are highly dysregulated in CSCs due to genetic changes [32, 33]. Among these pathways, STAT3 pathway has been identified as a key regulator of cancer stemness properties [34] and is involved in the ability of CSCs to survive, self-renewal, metastasize, evade the immune system, resist conventional cancer therapies, and lead to eventual cancer recurrence [35]. STAT3 is a functional marker for CSCs activity and a potential target for CSCs-directed therapy [32]. Small-molecules targeting STAT3 signaling in CSCs are becoming novel therapeutic strategies for HCC precision therapy [35]. Thus, the identification of natural small-molecule STAT3 inhibitors from medicinal plants is urgently needed for the development of drugs targeting HCC CSCs.

*cuspidatum* has been widely used in Traditional Chinese Medicines (TCM) to treat liver cancer and inflammations [36]. However, little has been known about how it works. In our previous work, 2-methoxystypandrone and two anthraquinone analogues such as emodin and physcion from *P. cuspidatum* have been also identified as STAT3 signaling inhibitors [14]. In the present study, the EtOAc extract of the roots of *P. cuspidatum* was further investigated and a novel juglone analogue 2-ethoxystypandrone (1) and a known compound citreorosein (2) were isolated and identified as STAT3 signaling inhibitors. It is interesting that 2-ethoxystypandrone (1) with ethoxyl (2-OCH_2_CH_3_) moiety at C-2 showed 2-fold increased potency compared with 2-methoxystypandrone with methoxyl (2-OCH_3_) group against STAT3 signaling with IC_50_ value of 7.75 ± 0.18 μM and 17.25 ± 0.21μM, respectively. These results suggested that the 2-OCH_2_CH_3_ group at C-2 might play important role in ligand binding to its molecular targets and is favorable for STAT3 signaling inhibitory activities. Our preliminary structure-activity relationship studies showed that α, β-unsaturated ketone group (para carbonyl groups) of 2-methoxystypandrone is the key pharmacophore for the inhibition of STAT3 signaling, and the phenolic group in the C-5 position of 2-methoxystypandrone is not essential to keep its bioactivities [14, 37]. 2-Ethoxystypandrone (1) might be considered as a potential STAT3 signaling inhibitor for developing an anti-cancer agent molecularly targeting CSCs in HCC. Future studies will focus on structural optimization study of 2-ethoxystypandrone (1).

STAT3 has been found to be constitutively activated in HCC cells and plays a pivotal role in oncogenesis, uncontrolled cell proliferation and cell survival, and inhibition of STAT3 activity demonstrated to induce apoptosis of HCC cells [27, 28]. In the present study, our experimental data demonstrated that 2-ethoxystypandrone (1) was able to block IL-6-induced STAT3 gene activation and the STAT3 phosphorylation, inhibit cell growth/cell survival and induce apoptosis of HCC cells. To examine the cytotoxicity of 2-ethoxystypandrone (1) against HCC cancer cell lines possessing different levels of basal activation of STAT3, we used a panel of HCC cells such as HepG2, HepG3B, SK-HEP-1, Li-7 and Huh-7, and found that 2-ethoxystypandrone (1) inhibited cell growth/survival more potently in human Li-7 and Huh-7 cancer cells (which contain constitutively activated STAT3, IC_50_ = 5.58 ± 0.89 μM and 3.69 ± 0.51μM, respectively) [27], as compared to those cells lacked aberrant STAT3 activation (IC_50_ = 16.71 ± 0.58 μM and 20.36 ± 2.90 μM, respectively). These results suggested that 2-ethoxystypandrone (1) could inhibit both IL-6-induced and basal activation of STAT3 in HCC cells, and significantly inhibit cell growth/survival of HCC cells, particularly those with constitutively activated STAT3 at the lower micromolar level. In our previous work, 2-methoxystypandrone, structurally similar to 2-ethoxystypandrone (1), exhibited anti-proliferative effects through inhibition of STAT3 and NF-κB pathways. Using a biotin-conjugated probe as a chemical tool, the direct cellular targets of 2-methoxystypandrone were then identified as Janus kinase 2 (JAK2) and IκB kinase (IKK), the upstream kinases of STAT3 and NF-κB signaling pathways, respectively [37]. 2-Methoxystypandrone was able to block STAT3 and NF-κB pathways by covalently interacting the upstream kinase JAK2 and IKK, inhibited cell growth/survival, and eventually induced apoptosis in human cancer cells [15, 37]. Since 2-ethoxystypandrone (1) is structurally similar to 2-methoxystypandrone, 2-ethoxystypandronec (1) might be proposed to have the similar molecular targets such as the upstream kinase JAK2 of STAT3 signaling pathway responsible for anti-proliferative effects in HCC cells.

STAT3 is a key transcriptional regulator of CSCs in HCC and plays an important role in self-renewal, cell proliferation, and cell apoptosis of CSCs in HCC [5]. Blocking STAT3 signaling might offer a novel opportunity to eradicate HCC CSCs [34]. In the present study, our experimental data showed that 2-ethoxystypandrone (1) significantly inhibited self-renewal and the tumorsphere formation of HCC CSCs isolated from HCC Huh-7 cells in a dose-dependant manner (Fig. 4a and b). The IC_50_ value of 2-ethoxystypandrone (1) was 2.70 ± 0.28 μM in the tumorsphere formation assay (Fig. 4c), whereas 2-Ethoxystypandrone (1) inhibited cell proliferation of HCC Huh-7 cells with an IC_50_ value of 3.69 ± 0.51 μM. 2-Ethoxystypandrone (1) displayed a little stronger cell proliferation inhibitory effect on HCC CSCs than parent HCC Huh-7 cells. According to recently reported studies, CSCs isolated from human tumors are predominantly (75%) in G0/G1 phases, indicating that most of CSCs are in a quiescent cell-cycle state [38]. In the present study, cell-cycle analysis showed that 61.65 ± 0.28% of HCC CSCs in control group were found to be a quiescent cell-cycle state in G0/G1 phases (Fig. 5b). After the treatment with 2-ethoxystypandrone (1) at 10 μM for 24 h, HCC CSCs significantly showed significant change in progress from a quiescent cell-cycle state in G0/G1(61.65 ± 0.28%), S phase (31.86 ± 1.07%), G2/M (6.48 ± 0.86%) to a cycling state with a significant increase in S (43.96 ± 1.07%) and G2/M (18.5 ± 0.36%) and subsequent decrease in G0/G1 phases (37.53 ± 1.32%), respectively. 2-Ethoxystypandrone (1) induced cell-cycle arrest of HCC CSCs in S and G2/M phases. Therefore, anti-proliferative activity of 2-ethoxystypandrone (1) against HCC CSCs might be caused by inducing an accumulation of cells in S phase and resulting in a G2/M block. Our experimental data showed that 2-ethoxystypandrone (1) was able to inhibit self-renewal and cell proliferation of HCC CSCs by blocking cell-cycle progression at the S and G2/M phases. Cell-cycle progression is tightly controlled by the sequential activation of the enzymes known as the cyclin-dependent kinases (CDKs) [39]. 2-Ethoxystypandrone (1) might affect cell-cycle regulators such as CDKs, which trigger apoptosis-related signaling pathways and cascade events that ultimately lead to cell apoptosis of HCC CSCs. Further mechanistic studies are needed to elucidate its cell-cycle arrest mechanisms. Beside targeting protein kinases such as CDKs and JAKs, 2-ethoxystypandrone (1) might be proposed to block self-renewal, cell survival and suppress the tumorsphere formation of HCC CSCs by targeting key downstream stemness genes such as Notch, c-Myc, Oct3/4, Sox2, and KIf4 of STAT3 signaling pathway [34], the direct cellular targets of 2-ethoxystypandrone (1) blocking CSCs activity are still unclear and its exact mechanisms of action will be further explored.

In the present study, we have focused on isolation, characterization and preliminary in vitro bio-activity studies of STAT3 signaling inhibitor 2-ethoxystypandrone (1) from *P. cuspidatum*. 2-Ethoxystypandrone (1) could be able to block STAT3 activity either via directly targeting STAT3 protein phosphorylation or by binding to the upstream tyrosine kinases such as JAKs in the STAT3 signaling pathway. Further investigations will be needed to identify the precise site of action of 2-ethoxystypandrone (1) against HCC CSCs.

## Conclusion

In summary, the EtOAc extract of the roots of *P. cuspidatum* was investigated and a novel juglone analogue 2-ethoxystypandrone (1) along with seven known compounds was further isolated. 2-Ethoxystypandrone (1) demonstrated a novel and potent small-molecule STAT3 signaling inhibitor, which strongly blocked STAT3 signaling, inhibited cell growth of HCC cells, blocked self-renewal, the tumorspheres formation, and induced apoptosis of HCC CSCs. 2-Ethoxystypandrone (1) may be considered as a novel STAT3 signaling inhibitor lead compound for developing anti-cancer agents targeting HCC CSCs. Further investigations are underway in our laboratory to develop more efficient derivatives, identify its precise target proteins and finally determine clinical potentials of 2-ethoxystypandrone (1) as a cancer stemness STAT3 signaling inhibitor.

## Additional files

Additional file 1: **Figure S1.** IR spectrum of 2-ethoxystypandrone. **Figure S2.** ESI-MS spectrum of 2-ethoxystypandrone **Figure S3.** HR-ESI-MS spectrum of 2-ethoxystypandrone **Figure S4.** ^1^H-NMR spectrum of 2-ethoxystypandrone **Figure S5.** ^1^H-^1^H COSY spectrum of 2-ethoxystypandrone **Figure S6.** ^13^C-NMR spectrum of 2-ethoxystypandrone **Figure S7.** DEPT 135-NMR spectrum of 2-ethoxystypandrone **Figure S8.** ^1^H-^13^C HSQC spectrum of 2-ethoxystypandrone **Figure S9.** ^1^H-^13^C HMBC spectrum of 2-ethoxystypandrone Spectroscopic Data of Compounds. (DOCX 481 kb)

## Abbreviations

CSCs: cancer stem cells
DMSO: dimethyl sulfoxide
EtOAc: ethyl acetate
HCC: hepatocellular carcinoma
HMBC: Heteronuclear multiple-bond correlation spectroscopy
HSQC: Heteronuclear single-quantum correlation spectroscopy
IR: Infra red
JAK2: Janus kinase 2
MeOH: methanol
MS: mass spectrometry
MTT: 3-[4,5-dimethyl-thiazol-2-yl]-2,5- diphenyl tetrazolium bromide
NMR: nuclear magnetic resonance spectroscopy
PE: petroleum ether
PI: Propidium Iodide
SDS-PAGE: sodium dodecyl sulfate polyacrylamide gel electrophoresis
STAT3: signal transducer and transcription factor
UV: Ultraviolet
