# Supplementary material for: 2-Ethoxystypandrone, a novel small-molecule STAT3 signaling inhibitor from Polygonum cuspidatum, inhibits cell growth and induces apoptosis of HCC cells and HCC Cancer stem cells
Source: BMC Complement Altern Med. 2019 Feb 1;19:38. doi: 10.1186/s12906-019-2440-9 (PMC6359800; doi:10.1186/s12906-019-2440-9)
Supplement: Supplementary file 1 — Figure S1. IR spectrum of 2-ethoxystypandrone. Figure S2. ESI-MS spectrum of 2-ethoxystypandrone Figure S3. HR-ESI-MS spectrum of 2-ethoxystypandrone Figure S4. 1H-NMR spectrum of 2-ethoxystypandrone Figure S5. 1H-1H COSY spectrum of 2-ethoxystypandrone Figure S6. 13C-NMR spectrum of 2-ethoxystypandrone Figure S7. DEPT 135-NMR spectrum of 2-ethoxystypandrone Figure S8. 1H-13C HSQC spectrum of 2-ethoxystypandrone Figure S9. 1H-13C HMBC spectrum of 2-ethoxystypandrone Spectroscopic Data of Compounds. (DOCX 481 kb) [file 12906_2019_2440_MOESM1_ESM.docx]

**Supplementary Materials**


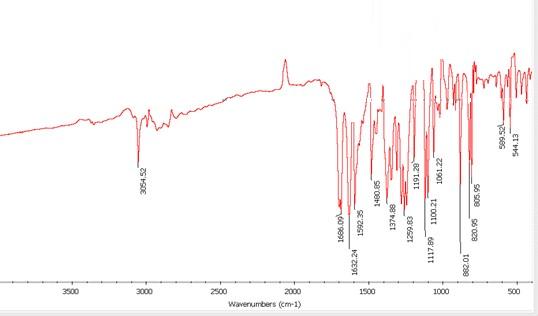


Figure S1. IR spectrum of 2-ethoxystypandrone (**1**).

Figure S2. ESI-MS spectrum of 2-ethoxystypandrone (**1**).

Figure S3. HR-ESI-MS spectrum of 2-ethoxystypandrone (**1**).


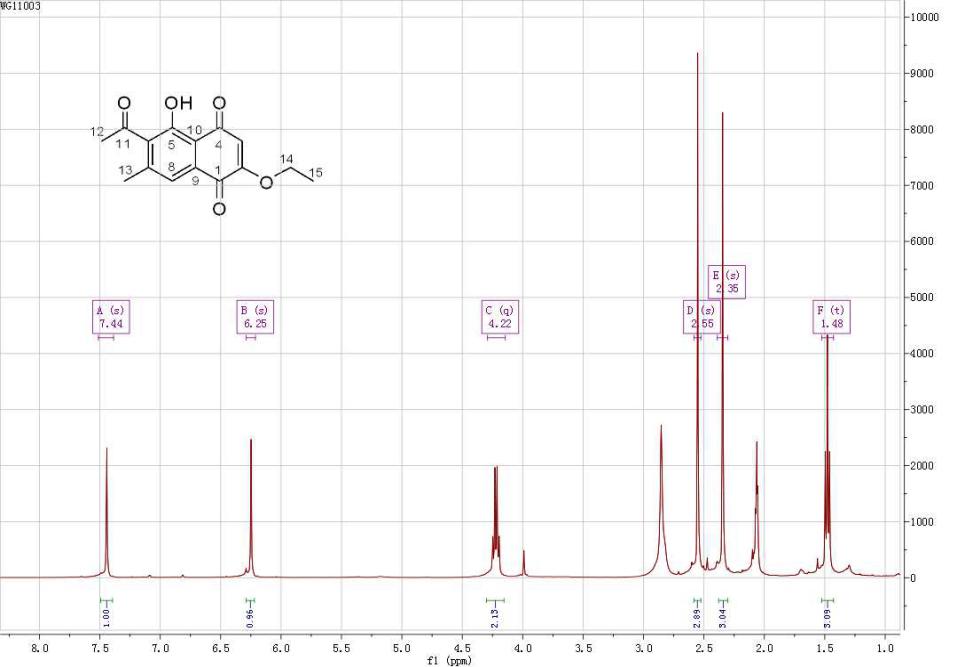


Figure S4. ^1^H-NMR spectrum of 2-ethoxystypandrone (**1**).


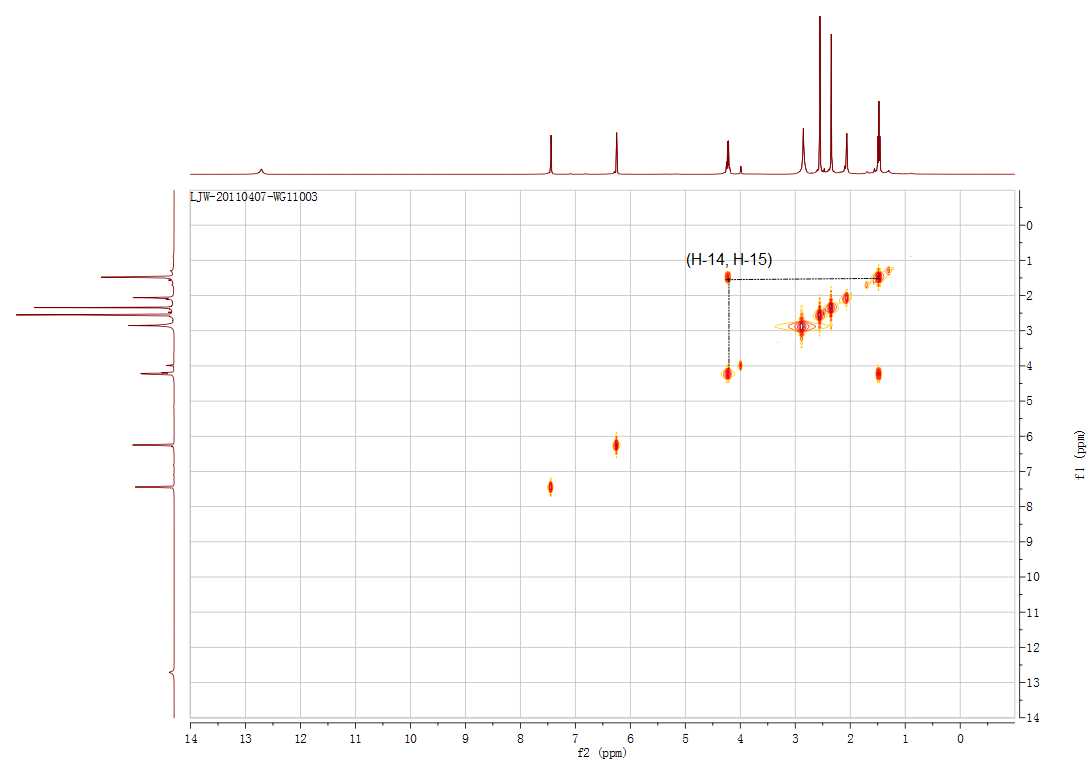


Figure S5. ^1^H-^1^H COSY spectrum of 2-ethoxystypandrone (**1**).


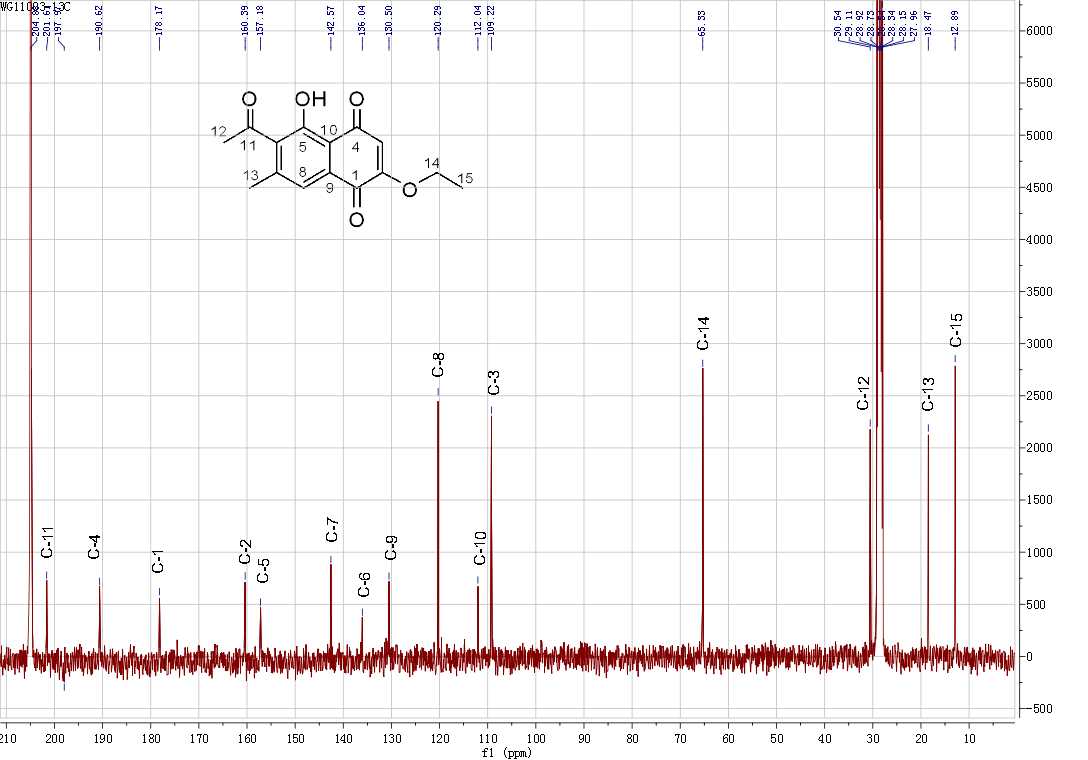


Figure S6. ^13^C-NMR spectrum of 2-ethoxystypandrone (**1**).


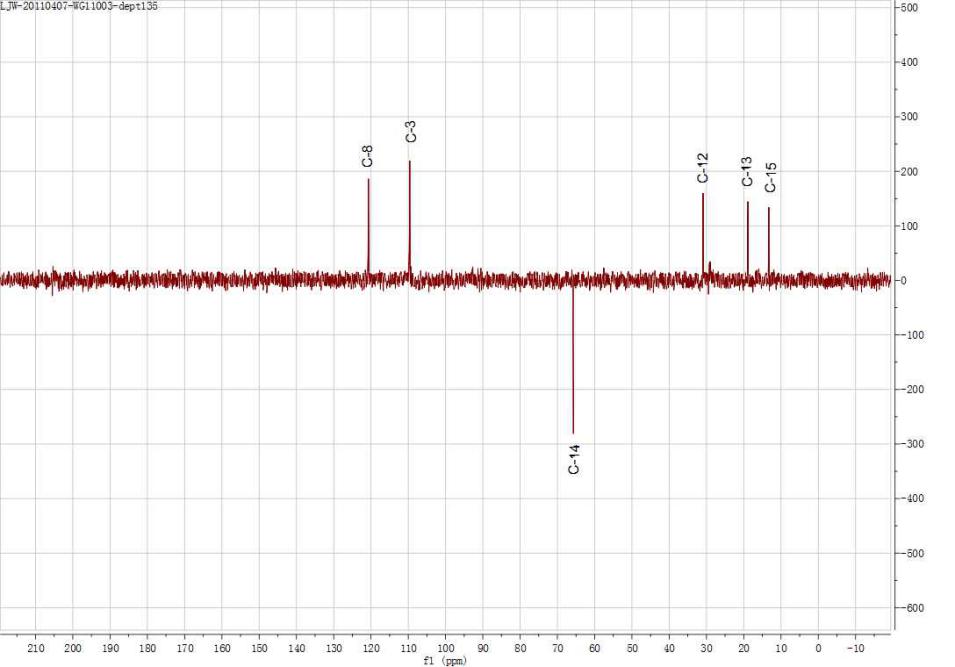


Figure S7. DEPT 135-NMR spectrum of 2-ethoxystypandrone(**1**).


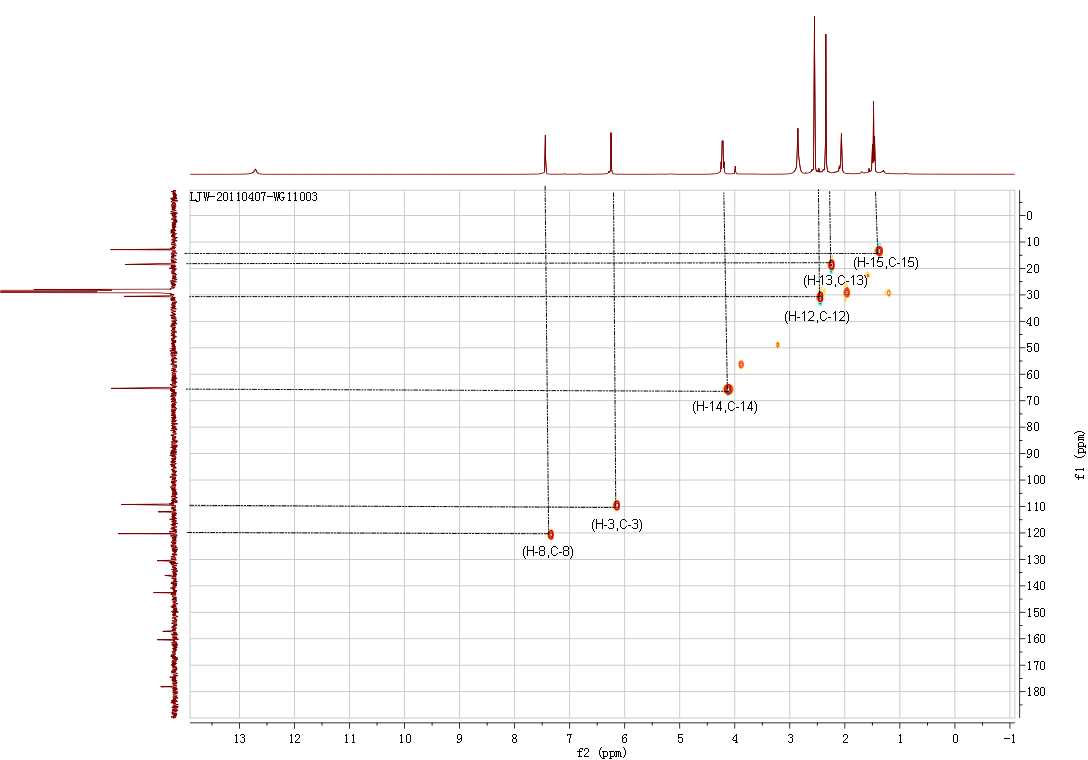


Figure S8. ^1^H-^13^C HSQC spectrum of 2-ethoxystypandrone (**1**).


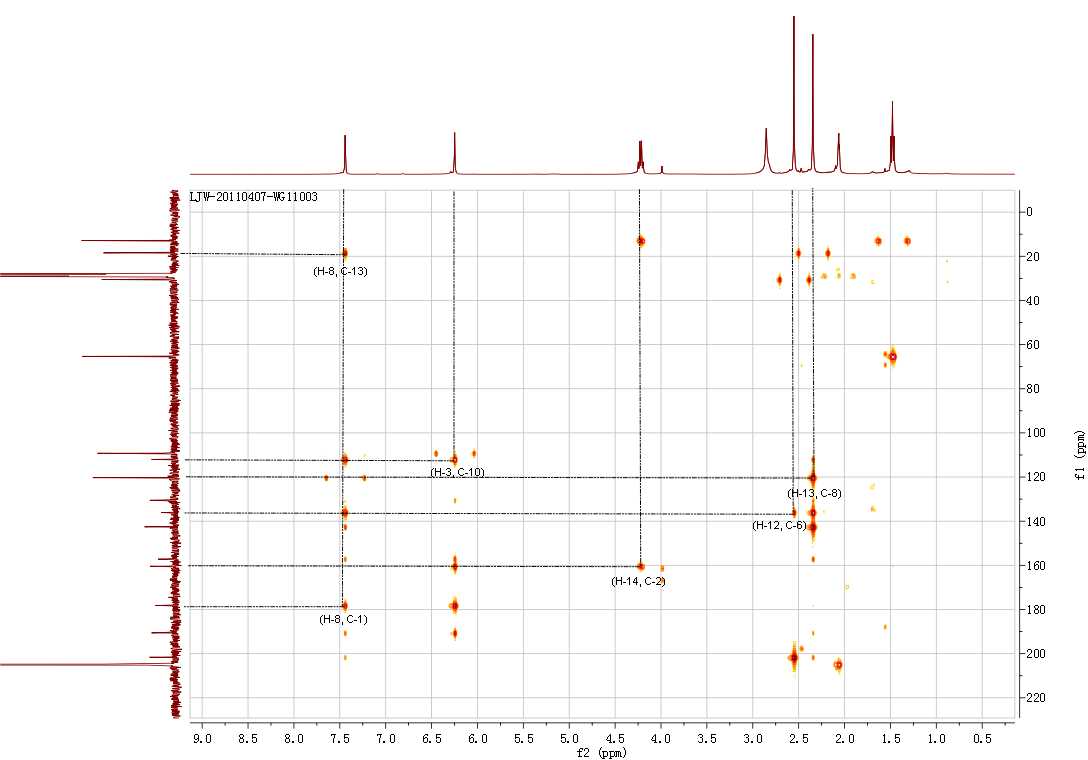


Figure S9. ^1^H-^13^C HMBC spectrum of 2-ethoxystypandrone (**1**).

**Spectroscopic Data of Compounds 2-8:**

Citreorosein (**2**): yellow needle crystal; ESI/MS: 284.84 [M-H]^-^; ^1^H-NMR (acetone-*d*_6_, 400 MHz) δ: 7.34 (1H, s, H-4), 7.11 (1H, s, H-5), 6.75 (1H, s, H-2), 6.48 (1H, s, H-7), 2.43 (3H, s, Ar-*C*H_2_) ; ^13^C-NMR (acetone-*d*_6_, 125 MHz) δ: 163.3 (C-1), 121.7 (C-2), 153.8 (C-3), 118.1 (C-4), 108.9 (C-5), 166.4 (C-6), 110.2 (C-7), 167.3 (C-8), 191.6 (C-9), 182.4 (C-10), 136.7 (C-10a), 134.5 (C-4a), 115.3 (C-9a), 109.7 (C-8a), 63.7 (CH_2_).

4,6-dihydroxybenzofuran-3-one (**3**): white amorphous powder; ESI/MS: 164.95 [M-H]^-^; ^1^H-NMR (acetone-*d*_6_, 400 MHz) δ: 5.23 (1H, s, H-2), 6.39 (1H, s, H-7), 6.55 (1H, s, H-8); ^13^C-NMR (acetone-*d*_6_, 100 MHz) δ: 70.3 (C-2), 171.4 (C-3), 151.5 (C-4), 103.0 (C-5), 158.6 (C-6), 101.9 (C-7), 166.1 (C-8), 104.3 (C-9).

Resveratrol (**4**): faint yellow needle crystal; ESI/MS: 227.18 [M-H]^-^; ^1^H-NMR (acetone-*d*_6_, 400 MHz) δ: 6.55 (1H, d, *J* = 2.1 Hz, H-2), 6.28 (1H, t, *J* = 2.1 Hz, H-4), 6.55 (1H, d, *J* = 2.1 Hz, H-6), 6.91 (1H, d, *J* = 16.3 Hz, H-7), 7.00 (1H, d, *J* = 16.3 Hz, H-8), 7.42 (1H, d, *J* = 8.5 Hz, H-10), 6.84 (1H, d, *J* = 8.5 Hz, H-11), 6.84 (1H, d, *J* = 8.5 Hz, H-13), 7.42 (1H, d, *J* = 8.5 Hz, H-14); ^13^C-NMR (acetone-*d*_6_, 100 MHz) δ: 140.9 (C-1), 105.7 (C-2), 159.6 (C-3), 102.7 (C-4), 159.6 (C-5), 105.7 (C-6), 126.9 (C-7), 129.1 (C-8), 130.0 (C-9), 128.7 (C-10), 116.4 (C-11), 158.2 (C-12), 140.9 (C-13), 105.7 (C-14).

Polydatin-2′-O-gallate (**5**): brown amorphous powder; ESI/MS: 541.18 [M-H]^-^; ^1^H-NMR (CD_3_OD, 400 MHz) δ: 6.60 (1H, s, H-1), 6.34 (1H, t, *J* = 2.0 Hz, H-3), 6.67 (1H, s, H-5), 6.81 (1H, d, *J* = 16.4 Hz, H-7), 6.92 (1H, d, *J* = 16.4 Hz, H-8), 7.33 (1H, d, *J* = 8.6 Hz, H-10), 6.77 (1H, d, *J* = 8.6 Hz, H-11), 6.77 (1H, d, *J* = 8.6 Hz, H-13), 7.33 (1H, d, *J* = 8.6 Hz, H-14), 5.16 ( 1H, d, *J* = 8.2 Hz, H-1′), 5.17 (1H, dd, *J* = 8, 8.2 Hz, H-2′), 3.80 (1H, dd, *J* = 7.9, 3.9 Hz, H-3′), 3.57 (1H, m, H-4′), 3.60 (1H, m, H-5′), 4.00 (1H, d, *J* = 10.8 Hz, H-6′a), 3.76 (1H, d, *J* = 8.4 Hz, H-6′b), 7.14 (1H, s, H-2′′), 7.14 (1H, s, H-6′′); ^13^C-NMR (CD_3_OD, 100 MHz) δ: 107.3(C-1), 159.5(C-2), 104.5(C-3), 158.4(C-4), 109.1(C-5), 140.0(C-6), 126.5(C-7), 130.3(C-8), 130.2(C-9), 129.0(C-10), 116.5(C-11), 160.4(C-12), 116.5(C-13), 129.0(C-14), 101.5(C-1′), 75.5(C-2′), 76.3(C-3′), 71.7(C-4′), 78.5(C-5′), 62.6(C-6′), 121.5(C-1′′), 110.5(C-2′′), 146.5(C-3′′), 141.0(C-4′′), 146.5(C-5′′), 110.5(C-6′′), 167.8(C-7′′).

Polydatin (**6**): white needle crystal; ESI/MS: 389.10 [M-H]^-^; ^1^H-NMR (CD_3_OD, 400 MHz) δ: 6.79 (1H, t, *J* = 3.4 Hz, H-2), 6.46 (1H, t, *J* = 2.0 Hz, H-4), 6.63 (1H, t, *J* = 2.0 Hz, H-6), 6.88 (1H, d, *J* = 16.3 Hz, H-7), 7.05 (1H, d, *J* = 16.3 Hz, H-8), 7.38 (1H, d, *J* = 8.3 Hz, H-10), 6.84 (1H, d, *J* = 8.5 Hz, H-11), 6.84 (1H, d, *J* = 8.5 Hz , H-13), 7.38(1H, d, *J* = 8.3 Hz, H-14), 4.91 (1H, d, *J* = 6.8 Hz, H-1′), 3.47 (1H, t, *J* = 8.8 Hz, H-2′), 3.41 (1H, m, H-3′), 3.45 (1H, t, *J* = 8.8 Hz, H-4′), 3.49 (1H, m, H-5′), 3.94 (1H, dd, *J* = 2.0 Hz, 12.0 Hz, H_a_-6′′), 3.73 (1H, dd, *J* = 5.7 Hz, 12.0 Hz, H_b_-6′′); ^13^C-NMR (CD_3_OD, 100 MHz) δ: 141.5 (C-1), 107.1 (C-2), 160.5 (C-3), 104.2 (C-4), 159.6 (C-5), 108.4 (C-6), 126.7 (C-7), 130.0 (C-8), 130.4 (C-9), 128.9 (C-10), 116.5 (C-11), 158.5 (C-12), 116.5 (C-13), 128.9 (C-14), 102.5 (C-1′), 75.0 (C-2′), 78.3 (C-3′), 71.5 (C-4′), 78.1(C-5′), 62.7 (C-6′) .

Catechin-3-O-gallate (**7**): brown amorphous powder; ESI/MS: 441.05 [M-H]^-^; ^1^H-NMR (CD_3_OD, 500 MHz) δ: 5.02 (1H, s, H-2), 5.52 (1H, s, H-3), 3.00 (1H, dd, *J* = 17.4 Hz, 4.5 Hz, H-4a), 2.85 (1H, dd, *J* = 17.4 Hz, 4.5 Hz, H-4b), 5.97 (1H, s, H-6), 5.97 (1H, s, H-8), 6.94 (1H, d, *J* = 1.5 Hz, H-12), 6.81 (1H, dd, *J* = 8.3 Hz, 1.6 Hz, H-15), 6.70 (1H, d, *J* = 8.2 Hz, H-16), 6.95 (1H, s, H-2′), 6.95 (1H, s, H-6′) ; ^13^C- NMR (CD_3_OD, 125 MHz) δ: 77.2 (C-2), 68.6 (C-3), 25.5 (C-4), 156.4 (C-5), 95.2 (C-6), 156.4 (C-7), 91.5 (C-8), 155.9 (C-9), 98.0 (C-10), 130.1 (C-11), 113.7 (C-12), 144.6 (C-13), 144.6 (C-14), 114.6 (C-15), 118.0 (C-16), 120.0 (C-1′), 108.8 (C-2′), 144.9 (C-3′), 138.4 (C-4′), 144.9 (C-5′), 108.8 (C-6′), 166.2 (C-7′) .

Torachrysone-8-O-*β*-D-glucopyranoside (**8**): faint yellow needle crystal; ESI/MS: 407.11 [M-H]^-^; ^1^H-NMR (CD_3_OD, 500 MHz) δ: 6.82 (1H, s, H-4), 7.00 (1H, s, H-5), 7.03 (1H, s, H-7), 1.99 (3H, s, CH_3_), 5.10 (1H, d, *J* = 7.7 Hz, H-1′), 3.54 (1H, m, H-2′), 3.45 (1H, m, H-3′), 3.50 (1H, m, H-4′), 3.56 (1H, m, H-5′), 3.76 (1H, dd, *J* = 12.2 Hz, 2.0Hz, H_a_-6′), 3.95 (1H, dd, *J* = 12.2 Hz, 5.7 Hz, H_b_-6′); ^13^C-NMR (CD_3_OD, 125 MHz) δ: 155.7 (C-1), 122.5 (C-2), 137.8 (C-3), 119.0 (C-4), 102.8 (C-5), 168.0 (C-6), 103.0 (C-7), 159.0 (C-8), 108.9 (C-9), 134.11 (C-10), 207.2 (C-11), 101.1 (C-1′), 73.5 (C-2′), 76.7 (C-3′), 69.8 (C-4′), 77.4 (C-5′), 60.9 (C-6′) , 37.3 (CO*C*H_3_) , 54.6 (O*C*H_3_) , 18.9 (Ar-*C*H_3_).
